# New Insights into the Role of Mitochondrial Dynamics and Autophagy during Oxidative Stress and Aging in the Heart

**DOI:** 10.1155/2014/210934

**Published:** 2014-07-15

**Authors:** Yoshiyuki Ikeda, Sebastiano Sciarretta, Narayani Nagarajan, Speranza Rubattu, Massimo Volpe, Giacomo Frati, Junichi Sadoshima

**Affiliations:** ^1^Cardiovascular Research Institute, Department of Cell Biology and Molecular Medicine, Rutgers New Jersey Medical School, 185 South Orange Avenue, Medical Science Building G-609, Newark, NJ 07103, USA; ^2^IRCCS Neuromed, Via Atinense 18, 86077 Pozzilli, Italy; ^3^Division of Cardiology, Department of Clinical and Molecular Medicine, Faculty of Medicine and Psychology, Sapienza University, Via di Grottarossa 1035-1039, 00189 Rome, Italy; ^4^Department of Medico-Surgical Sciences and Biotechnologies, Sapienza University, Corso della Repubblica 79, 04100 Latina, Italy

## Abstract

The heart is highly sensitive to the aging process. In the elderly, the heart tends to become hypertrophic and fibrotic. Stiffness increases with ensuing systolic and diastolic dysfunction. Aging also affects the cardiac response to stress. At the molecular level, the aging process is associated with accumulation of damaged proteins and organelles, partially due to defects in protein quality control systems. The accumulation of dysfunctional and abnormal mitochondria is an important pathophysiological feature of the aging process, which is associated with excessive production of reactive oxygen species. Mitochondrial fusion and fission and mitochondrial autophagy are crucial mechanisms for maintaining mitochondrial function and preserving energy production. In particular, mitochondrial fission allows for selective segregation of damaged mitochondria, which are afterward eliminated by autophagy. Unfortunately, recent evidence indicates that mitochondrial dynamics and autophagy are progressively impaired over time, contributing to the aging process. This suggests that restoration of these mechanisms could delay organ senescence and prevent age-associated cardiac diseases. Here, we discuss the current understanding of the close relationship between mitochondrial dynamics, mitophagy, oxidative stress, and aging, with a particular focus on the heart.

## 1. Introduction

Over the past few decades, the human life span has been extended and is expected to increase further in the next few years with a supplementary prolongation of life expectancy. Consequently, the prevalence of age-related diseases, such as cardiovascular diseases (CVD), is increasing [1]. Hypertension, dyslipidemia, diabetes mellitus, and metabolic syndrome increase with age and play a key role in the development of CVD, which is a major cause of chronic disability, morbidity, and mortality in the elderly [2, 3]. The aging process is associated with accumulation of damaged proteins and organelles that is partly attributable to a decline in the maintenance of protein quality control systems [4–6]. In addition, a critical pathological feature of the aging process is the development of mitochondrial abnormalities. Reactive oxygen species (ROS) progressively accumulate during aging as a result of impairment of mitochondrial oxidative phosphorylation, misfolded protein accumulation, and an imbalance between the expression of oxidant and antioxidant proteins [7–11]. ROS accumulation, in turn, further attenuates the bioenergetic function of mitochondria by causing mutations in mitochondrial DNA (mtDNA), impairing the tricarboxylic acid cycle (TCA) and the electron transport chain complexes. This causes a vicious cycle that further and progressively aggravates oxidative stress in tissues and mitochondrial dysfunction in aged organs [9–11]. Therefore, an optimally functioning regulation system that is able to eliminate damaged cellular constituents and organelles, in particular mitochondria, is critical to maintain cellular homeostasis during aging. As recently highlighted, cardiomyocytes are not terminally differentiated cells and have the capacity to reenter the cell cycle, but this process seems to be quenched by a limited turnover, making them reliant on these mechanisms to remove damaged or long-lived cellular components or proteins [12, 13].

Mitochondrial dynamics, particularly mitochondrial fusion and fission, are important processes for mitochondrial homeostasis [14, 15]. In particular, mitochondrial fission allows for selective elimination of damaged mitochondria by autophagy [16, 17]. Autophagy, which is derived from the Greek words “*aut* $\overset{`}{o}$ *s*” (*α* *ὐ* *τ* $\overset{´}{o}$ *ς*, for self) and “*phagèin”* (*φ* *ά* *γ* *ω*, for eating), is an evolutionarily conserved system that targets damaged or long-lived proteins and organelles for degradation through lysosomes [16, 18]. Autophagy can be either nonselective or cargo-specific. Mitochondrial-specific autophagy is referred to as mitophagy. Accumulating lines of evidence indicate that autophagy and mitophagy play a crucial role in the regulation of cardiac homeostasis and response to stress [17–19].

It is now known that abnormalities in mitochondrial dynamics and autophagy contribute to the aging process [14–16]. This review will deal with the current understanding concerning the close relationship between mitochondrial dynamics, mitophagy, oxidative stress, and aging in the heart.

## 2. Oxidative Stress, Mitochondria, and Aging

Oxidative stress is reported to increase in hypertension, hyperlipidemia, diabetes mellitus, and smoking, where it contributes to the development of atherosclerosis [20]. Oxidative stress is also known to be involved in the development of cancer and Alzheimer's disease associated with aging [21, 22]. ROS are constantly generated in cells as a byproduct of oxygen metabolism [9] since they are important cell signaling molecules at physiological levels [23]. For instance, we recently demonstrated that ROS production by Nox4, a member of the NADPH oxidase family, in the endoplasmic reticulum is important for autophagy induction and cell survival during cardiomyocyte energy deprivation [24]. However, excessive ROS induce an imbalance between the oxidant and antioxidant responses in cells [9].

Oxidative stress is increased during the organ aging process [9, 11, 25]. Production of ROS is progressively enhanced over the years in different organelles. We have demonstrated that mitochondrial expression of Nox4 is upregulated in the senescent heart, contributing to ROS production and development of cardiac abnormalities [26]. In fact, mice with cardiac-specific overexpression of Nox4 develop cardiomyopathy in the late phase of life [26]. Recent evidence from lower organisms also indicates that the homolog of Nox4 in yeast is upregulated in the endoplasmic reticulum in response to mitochondrial dysfunction [27], a mechanism that would contribute to the aging process. P66 adaptor protein also accumulates progressively in aged mitochondria, and p66 deletion significantly extends life span in mammals [28]. ROS favor accumulation of misfolded proteins that, in turn, further enhance oxidative stress [29]. Of note, ROS not only damage proteins by directly oxidizing them but also have been shown to impair the activity of immunoglobulin heavy chain binding protein (Bip) and protein disulfide isomerase (PDI) in the endoplasmic reticulum of the mouse liver, thereby affecting the protein folding process during aging [30].

Antioxidant gene expression and activity are also affected during aging. The capacity for activating manganese superoxide dismutase (MnSOD) and catalase expression during oxidative stress and exercise is reduced over time in skeletal muscle [31]. In addition, reduction of sirtuins (SIRT) promotes ROS production during aging. SIRT3 deacetylates and activates MnSOD in the mouse liver [32]. SIRT3 also increases the activity of isocitrate dehydrogenase 2 during aging, thereby stimulating the tricarboxylic acid (TCA) cycle in the ear system, liver, and brain in mouse [33]. This event increases the amount of mitochondrial NADPH and protects against oxidative-stress-induced damage by increasing the ratio of reduced-to-oxidized glutathione. SIRT1 also promotes antioxidant defense through the activation of Forkhead box protein O1 (FOXO1) signaling in multiple mammalian cell lines [34]. The SIRT3 expression level was found to be reduced in aged skeletal muscle [35]. Although SIRT1 expression seems to increase in the elderly [36], ROS were found to be able to promote SIRT1 degradation through its oxidation in lung epithelial cells [37]. Furthermore, SIRT1 activity is reduced during aging due to a dramatic reduction of NAD+ levels in mouse skeletal muscle [38]. Of note, ROS-dependent AMP-activated protein kinase (AMPK) inhibition could also potentially lead to a reduction of antioxidant defenses during aging. In fact, AMPK can stimulate the antioxidant response through FOXO1 activation [39]. We recently found that ROS can directly oxidize AMPK at cysteines 130 and 174 and inhibit its activity in cardiomyocytes [40].

In apparent contrast with this evidence, it has been shown that impaired insulin/insulin growth factor 1 (IGF-1) signaling and deletion of superoxide dismutase 2 extend life span in* C. elegans *through a mild increase in oxidative stress. This low accumulation of ROS is not sufficient to promote cellular damage, whereas it is able to trigger several cellular adaptive responses that are able to increase the organism's resistance to later stronger stresses [41, 42]. Therefore, despite the fact that pathological increases in ROS are maladaptive and reduce longevity, transient and physiological ROS activate adaptive signaling pathways that can promote survival.

Mitochondrial dysfunction represents a common feature of the aging process [43]. ROS progressively promote mtDNA mutations and deletions that accumulate over time, leading to progressive reduction of the number of mtDNA copies [44, 45]. Oxidative damage of mtDNA contributes to impairment of the electron transport chain, to mitochondrial uncoupling, and, ultimately, to bioenergetic dysfunction of mitochondria characterized by reduced ATP production and further accumulation of ROS [46, 47]. In support of the importance of mtDNA mutations in the aging process, it has been observed that a mouse model with defective mtDNA polymerase (PolgA) displays a high rate of mtDNA mutations and a reduced life span, which is associated with early signs of senescence such as osteoporosis, hair and weight loss, anemia, and cardiac hypertrophy [48]. In addition to damaging mtDNA, ROS can directly impair the TCA cycle, particularly by targeting aconitase [49]. ROS can also impair the enzymes involved in oxidative phosphorylation, particularly the enzymes containing an iron-sulphur cluster, in eukaryotic cells [50]. Finally, oxidative stress is associated with mitochondrial transition pore opening, which promotes necrosis and cell death in mammalian cells [51]. The observation that systemic mitochondrial overexpression of catalase extends life span and delays the aging process in mice is evidence that mitochondrial ROS strongly contribute to the aging process [52]. In addition, mitochondrial catalase prevents age-associated mitochondrial dysfunction and insulin resistance [53]. Of note, overexpression of catalase in subcellular organelles other than mitochondria failed to extend life span in mice, and overexpression of different isoforms of superoxide dismutase and peroxidase enzymes was not associated with a significant extension of longevity [54, 55]. These results suggest that accumulation of ROS, more specifically hydrogen peroxide, in mitochondria, but not in other organelles, contributes to the aging process in mammals. This is likely due to a progressive reduction of mitochondrial function [54, 55]. It should be pointed out that a reduction of sirtuin activity during aging can also contribute to mitochondrial dysfunction independently of ROS. For example, SIRT3 can stimulate oxidative phosphorylation by directly activating the electron transport chain complexes in the heart, kidney, and liver [56]. SIRT3 deletion can also accelerate age-dependent cardiac hypertrophy by the lack of deacetylation of cyclophilin D at lysine 166 [57]. This mechanism prevents age-dependent mitochondrial transition pore opening and ROS accumulation.

Mitochondrial abnormalities and dysfunction play a fundamental pathologic role during the aging process in the heart [58, 59]. This is not surprising based on the evidence that the heart is an organ that relies on extensive energy production to support the cardiac cycle. Enlarged mitochondria with membrane and matrix abnormalities accumulate in the aged heart [58, 59]. Mitochondrial ROS production and mtDNA oxidative damage also progressively accumulate [58, 59]. As a result, increased mitochondrial ROS can promote cardiac hypertrophy and cardiac fibrosis [58, 59]. We previously showed that aging upregulates Nox4 [26] and that upregulation of Nox4 in the nucleus promotes cardiac hypertrophy through oxidation and nuclear export of histone deacetylase 4 (HDAC4) [60]. ROS accumulation also promotes cardiac inflammation, with increased recruitment of inflammatory cells, fibroblast activation, and, ultimately, hypertrophy and fibrosis [61]. Diastolic dysfunction is highly prevalent in the aged heart [58, 59] due to increased fibrosis as well as direct oxidation and inhibition of sarcoplasmic reticulum Ca 2+ ATPase (SERCA2a) [62] and reduction of mitochondrial function [63]. Systolic dysfunction may also occur during aging, through mitochondrial-dysfunction-dependent apoptosis and impairment of energy production [58, 59, 63, 64].

Therefore, it appears clear that the mechanisms that guarantee quality control in mitochondria may play a fundamental role during the aging process by limiting the accumulation of damaged mitochondria and organ damage. Recently, it has been reported that mitochondrial fusion and fission represent important mechanisms for preserving mitochondrial function [14]. In fact, fusion improves mitochondrial oxidative capacity and limits age-dependent mtDNA mutations, whereas fission allows the maintenance of functional mitochondria by segregating damaged parts. Importantly, damaged mitochondria undergo quality control through selective elimination by mitophagy [17].

## 3. Autophagy and Cardiac Aging

Autophagy plays a crucial role in the degradation of long-lived proteins and organelles. These properties make autophagy a crucial mechanism for maintaining tissue homeostasis during the aging process [16, 18]. Three types of autophagy have been identified: microautophagy, chaperone-mediated autophagy, and macroautophagy (commonly referred to as autophagy). Microautophagy is characterized by the direct delivery of sequestered cellular constituents into lysosomes. In microautophagy, the lysosomal membrane invaginates cytosolic components [16, 18]. In chaperone-mediated autophagy, chaperones such as heat-shock proteins help deliver macromolecules to the lysosomes. In macroautophagy, hereafter referred to as autophagy, a small vesicular sac, called the isolation membrane or phagophore, is initially formed. The phagophores enclose cytosolic long-lived proteins and organelles, resulting in the formation of double-membraned structures called autophagosomes. The autophagosomes then fuse with lysosomes, which leads to the degradation of the sequestered cellular constituents through digestion of the cargo by lysosomal hydrolases [16, 18].

**Figure 1:**
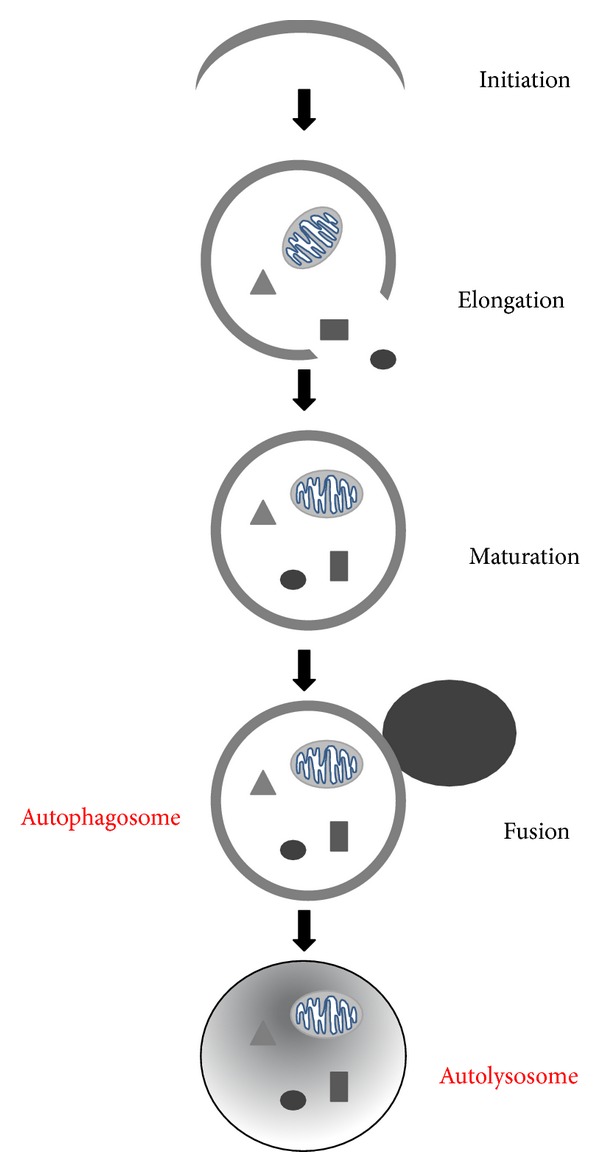
Autophagy machinery. Schema representing the four stages of the autophagic process.

The autophagic process consists of the following stages: initiation/nucleation, elongation, and maturation/retrieval of autophagosomes (Figure 1) [16–18, 65]. These processes require autophagy-related gene (Atg) proteins that are evolutionarily conserved and were initially identified in yeast [66].

Autophagy is deeply involved in the regulation of cardiac homeostasis and response to stress [19, 67–69]. Energy deprivation is a strong stimulus for autophagy. In response to nutrient deprivation, hypoxia and ischemia, autophagy is upregulated to digest damaged proteins and organelles, a process that also recycles amino acids for energy production, new enzyme synthesis, and, ultimately, maintenance of cellular functions [19, 67–69].

Autophagy is a critical mechanism for the aging process. Inhibition of autophagic proteins, such as Atg1, Atg7, Atg18, and Beclin1, significantly shortens life span in lower organisms [70]. In mammals, deletion of autophagic proteins in organs, such as the liver [71, 72], brain [73, 74], pancreas [75], and heart [76], is associated with early signs of senescence and dysfunction characterized by intracellular misfolded protein accumulation and the presence of aberrant mitochondria. On the other hand, whether autophagy activation is sufficient to extend life span in mammals is still under investigation. Inhibition of mTORC1 [77] and caloric restriction [78], two conditions that have been shown to enhance longevity, strongly promote autophagy [16]. In* Drosophila*, the protective effect of rapamycin on aging is abolished by concomitant inhibition of autophagy by Atg5 knockdown [79]. Interestingly, autophagy declines with age, and the impairment of autophagy contributes to neurodegeneration, insulin resistance, cardiac hypertrophy and dysfunction, and osteoarthritis [16]. Autophagy inhibition further worsens mitochondrial abnormalities and dysfunction and aggravates the accumulation of misfolded proteins [16]. This evidence suggests that autophagy inhibition during aging may play a significant causative role in age-associated diseases.

Accumulation of ROS and mitochondrial dysfunction appears to contribute to autophagy inhibition during aging [16]. In fact, ROS promote accumulation of oxidized proteins that tend to aggregate together in lysosomes. This accumulation of misfolded proteins further promotes ROS production, causing membrane lipid peroxidation and aggravating protein aggregation. When mitochondria are sequestered by lysosomes or are severely damaged and release their contents, mitochondrial peroxides accumulate in lysosomes and create a Fenton reaction that promotes crosslinking reactions among the accumulated misfolded proteins, resulting in formation of the pigment lipofuscin [80]. Lipofuscin accumulates in lysosomes and inhibits lysosomal activity [81]. As a result, autophagic flux is likely inhibited. The lysosome-associated membrane protein 2a (LAMP2a) is also downregulated during the aging process, contributing to the impairment of autophagic flux [82]. Of note, reactivation of LAMP2a restores both chaperone-mediated and conventional autophagy [82]. Also of note, multiple genes involved in autophagosome formation are reduced during aging, suggesting that the autophagy process is impaired at multiple levels [16].

Autophagy is inhibited in the aged heart [76]. It has been shown that mice with cardiac-specific deletion of Atg5 protein, through constitutive *αMHC-CRE* expression, develop a dilated cardiomyopathy during aging, characterized by severe systolic dysfunction, sarcomeric disarray, and accumulation of dysfunctional and abnormal mitochondria [76]. This evidence indicates that autophagy is required for cardiac homeostasis during aging, and autophagy inhibition contributes to cardiac senescence. Rapamycin and caloric restriction, both of which can reactivate autophagy, were shown to reverse age-dependent cardiac hypertrophy and diastolic dysfunction [83–86].

## 4. Mitochondrial Elimination by Autophagy

**Figure 2:**
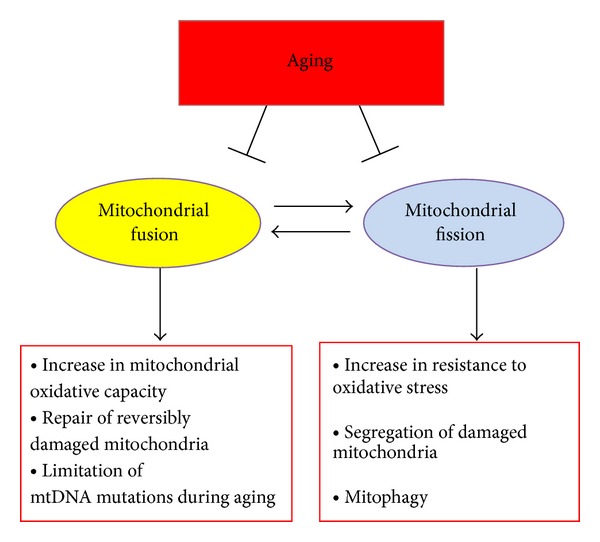
Mitochondrial dynamics. Schema explaining the main functions of mitochondrial fusion and fission.

Mitochondrial quality control is essential for maintaining tissue homeostasis [14, 15]. Mitochondrial quality control is regulated by the balance between the elimination of mitochondria through autophagy and mitochondrial biogenesis [14, 15]. Mitochondria are dynamic organelles that are constantly undergoing fission and fusion to adapt toward changes in the cellular environment. Fission produces small spherical mitochondria, whereas fusion produces tubular or elongated mitochondria [14, 15]. It is well established that mitochondrial dynamics play a crucial role in the quality control of mitochondria. Fusion of mitochondria that are reversibly damaged with healthy mitochondria can favor their functional repair. In addition, fusion can limit the accumulation of mtDNA mutations during aging [14, 15]. However, when mitochondria incur irreversible damage, fission leads to elimination of the damaged organelles (Figure 2) [87]. Mitochondrial dynamics are regulated by several different GTPases. Mitofusins 1 and 2 (Mfn1 and Mfn2) and optic atrophy 1 (Opa1) induce mitochondrial fusion in the outer and inner mitochondrial membranes, respectively [88, 89], while dynamin-related protein 1 (Drp1) is a cytoplasmic protein that assembles into rings surrounding the mitochondrial outer membrane, where it interacts with fission protein 1 (Fis1) to promote fission [90, 91]. As mentioned above, mitochondrial fusion and fission are required for the regulation of cardiac homeostasis and adaptation to stress [14, 15]. Mice with cardiac Mfn2 deletion develop cardiac hypertrophy associated with systolic dysfunction and mitochondrial dysfunction [92]. It was also found that Mfn2 mediates the differentiation of embryonal stem cells into cardiomyocytes and cardiac embryogenesis through calcineurin and Notch signaling [93]. On the other hand, Mfn2 overexpression promotes apoptosis in cardiomyocytes exposed to oxidative stress [94]. Mice with systemic heterozygous deletion of Opa1 develop pronounced hypertrophy and dysfunction in response to pressure overload [95]. Interestingly, inhibition of either Mfn1 or Mfn2 promotes generation of fragmented mitochondria, which inhibit cardiomyocyte mitochondrial transition pore opening and confer protection in response to oxidative stress [92, 96]. This data indicates that integrity of mitochondrial fusion is required for cardiomyocyte homeostasis and maintenance of cardiac function at baseline conditions. However, during stress, accumulation of small mitochondria is protective against ROS accumulation in cardiomyocytes. Functional mitochondrial fission is also crucial for preservation of cardiac structure and function. In fact, cardiac-specific deletion of Drp1 is associated with the development of cardiac dilation and dysfunction over time, which is related to inhibition of mitochondrial fission and autophagy (Ikeda Y and Sadoshima J, unpublished data).

Mitochondrial fission is an essential requirement for mitophagy [97]. Inhibition of fission results in disruption of mitophagy and accumulation of dysfunctional mitochondria [87]. The fragments which result from fission of mitochondria can differ in membrane potential (Δ*ψ*m) and fragments with low Δ*ψ*m are more likely to be targeted by mitophagy [87]. Oxidative stress can lead to this formation of asymmetrical daughter mitochondria characterized by different Δ*ψ*m [87]. In addition, increased mitochondrial pore opening promotes mitophagy through cyclophilin-D-dependent mechanisms, as observed in cardiomyocytes [98].

**Figure 3:**
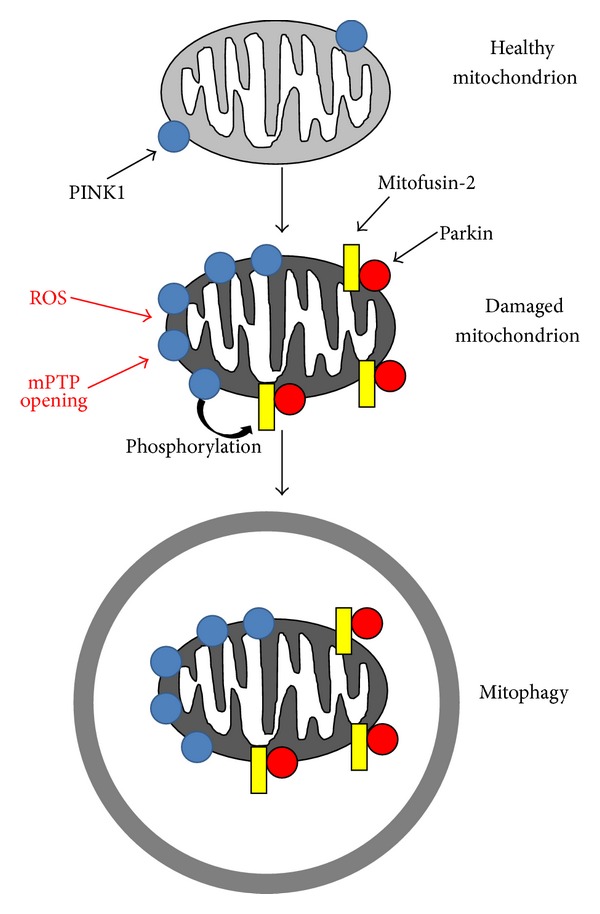
Mechanisms of mitophagy induction. Schema representing the main mechanisms of mitophagy induction. PINK1 accumulates in damaged mitochondria and phosphorylates mitofusin-2. Phosphorylated mitofusin-2 recruits parkin, which ubiquitinates several mitochondrial targets. Mitochondrial ubiquitination by parkin promotes mitophagy.

Several specific proteins have been reported to play a key role in the molecular regulation of mitophagy. PTEN-induced kinase 1 (PINK1) and parkin are associated with familial Parkinson's disease and have recently been identified as important regulators of mitophagy [97]. PINK1 is a serine/threonine kinase that is localized in mitochondria. Upon mitochondrial damage and loss of inner mitochondrial membrane potential, PINK1 is stabilized, accumulates, and promotes the recruitment of parkin in damaged mitochondria [99]. Parkin is a cytosolic E3-ubiquitin ligase that accumulates in depolarized mitochondria and promotes their clearance by mitophagy [100]. Parkin ubiquitinates several targets in damaged mitochondria, including voltage-dependent anion channels (VDAC) and Mfn2, and recruits ubiquitin-binding deacetylase HDAC6 and p62/sequestosome-1, which interact with LC3 and promote assembly of the autophagosome and, ultimately, removal of the damaged mitochondria (Figure 3) [101–103]. Of note, double deletion of Mfn1 and Mfn2 in the adult heart promotes cardiac dysfunction and mitochondrial fragmentation without associated mitophagy, suggesting that Mfn1 and Mfn2 are important for the mitophagic mechanism [104]. This hypothesis is supported by the recent demonstration that PINK1 phosphorylates Mfn2 at threonine 111 and serine 442 to promote its binding with parkin in damaged mitochondria, ultimately leading to mitophagic removal [105]. Aside from demonstrating the mechanisms through which PINK1 tags damaged mitochondria for subsequent recognition by parkin, this study strongly supports the existence of a tight relationship between mitochondrial dynamics and mitophagy. Furthermore, parkin and mitofusins have been demonstrated to cooperate in a balanced manner to regulate mitophagy and mitochondrial spheroid formation in response to ROS accumulation [106]. In addition, Mfn2 is involved in the regulation of autophagosome-lysosome fusion in cardiomyocytes [107].

Other specific proteins that have been reported to play a role in mitophagy are Bcl-2/adenovirus E1B 19-kDa–interacting protein-3 (Bnip3) and Nip3-like protein X (Nix) [97]. Both Nix and Bnip3 have been implicated in the pathogenesis of cancer and heart disease [108]. Nix is reported to be required for the selective elimination of mitochondria in erythrocytes in peripheral blood [109], while Bnip3 has been reported to induce hypoxia-mediated mitophagy downstream of hypoxia-inducible factor-1*α* [110]. Bnip3 was also shown to promote translocation of Drp1 in response to oxidative stress in cardiomyocytes [111], and Nix-dependent mitochondrial depolarization promotes mitophagy [108]. In addition, Nix interacts directly with LC3 and recruits the autophagosome to damaged mitochondria, thus acting as a receptor for autophagosome recruitment in this context [112].

Autophagic activity is reduced during aging [16]. As a direct consequence, mitophagy is also inhibited [16]. Mitochondrial dynamics are also altered during aging. Drp1 and Mfn2 expression levels are reduced in aged skeletal muscle [113]. In addition, peroxisome proliferator-activated receptor gamma coactivator (PGC)-1*α* levels are reduced in the skeletal muscle of the elderly [114]. PGC-1*α* promotes mitochondrial biogenesis and fusion by upregulating the levels of Mfn2 in human skeletal muscle [115]. Fis1 overexpression reduces cellular senescence in hepatic cells, suggesting that activation of fission offsets the aging process [116]. Abnormalities in mitochondrial dynamics and mitophagy significantly contribute to the accumulation of aberrant and dysfunctional mitochondria with progressive accumulation of mtDNA mutations.

Therefore, mitophagy is critical for turnover of whole damaged mitochondria. However, other cellular mechanisms promoting degradation of damaged mitochondrial proteins are also crucial for mitochondrial quality control [117, 118]. Damaged and misfolded mitochondrial proteins can be cleared by mitochondrial chaperones and proteases [117, 118]. Accumulating lines of evidence also indicate that the ubiquitin-proteasome system is critical for regulation of mitochondrial quality control [117, 118]. Inhibition of this system results in the accumulation of damaged and dysfunctional mitochondria [119]. In addition, defects of the ubiquitin-proteasome system result in cardiac hypertrophy and dysfunction and defective cardiac responses to stress [120, 121]. Multiple mitochondrial proteins have been shown to undergo ubiquitination and accumulate when proteasome is inhibited. Upon mitochondrial damage, parkin was shown to mediate the proteasomal degradation of Mfn 1 and 2 [122]. VDAC and Tom20 were also found to be potential targets of the ubiquitin-proteasome system [122]. Intriguingly, proteasomal degradation appears to also regulate the turnover of the inner mitochondrial membrane uncoupling protein 2 [123] and of the mitochondrial matrix oligomycin sensitivity conferral protein (OSCP) [124]. This data suggests the existence of complex mechanisms exposing the damaged inner membrane and matrix proteins to the ubiquitin-proteasome system. Interestingly, proteasomal degradation of mitochondrial proteins was shown to be tightly connected to the regulation of mitochondrial dynamics and mitophagy [117, 118]. Mfn1, Drp-1, and Fis1 can be degraded by the ubiquitin-proteasome system, thereby affecting fusion and fission [122, 125–127]. As described above, mitochondrial protein ubiquitination by parkin is required for mitophagy induction [122, 125, 127]. Intriguingly, it has been recently demonstrated that proteins regulating proteasome activity are also deeply involved in the regulation of the autophagic process [128]. In addition, a cross-talk between ubiquitin-proteasome system and autophagy has been demonstrated in cardiomyocytes* in vitro* and* in vivo *[129]. This data suggests an interplay between the ubiquitin-proteasome system and autophagy, which may share common mechanisms of regulation.

## 5. The Role of Autophagy in Cardiac Ischemia, Remodeling, and Hypertrophy

Cardiac aging is associated with increased susceptibility to ischemic injury and remodeling [45, 46]. Autophagy and mitochondrial dynamics play an important role in regulating cardiac adaptation to stress [14, 15, 58, 59]. Therefore, age-dependent abnormalities in autophagy and mitochondrial dynamics might be involved in the increased fragility of the aged heart during stress.

Cardiac ischemia decreases cellular ATP content, which leads to energy stress and abnormal ROS production associated with mitochondrial dysfunction [130]. Autophagy activation in the ischemic heart protectively counterbalances these mechanisms [16–18, 65]. Our group has recently elucidated the mechanisms through which autophagy is upregulated during energy deprivation. We found that activation of AMPK [131] and glycogen synthase kinase (GSK)-3*β* [132] as well as inhibition of Ras homolog enriched in brain (Rheb) [133] contributes to autophagy activation through the inhibition of mTORC1. Nox4-dependent production of ROS in the endoplasmic reticulum is also an important signaling event for induction of autophagy during energy deprivation through the activation of PERK signaling [24]. In addition, starvation activates FOXO1 through SIRT1-mediated deacetylation, which induces expression of Rab7 and stimulates autolysosome formation and autophagic flux [134].

Autophagy inhibition increases ischemic injury. Inhibition of endogenous AMPK during prolonged ischemia causes suppression of autophagy, accompanied by enlargement of the myocardial infarct [131]. Similarly, inhibition of GSK-3*β* and activation of Rheb enhance ischemic injury during ischemia [132, 133]. Interestingly, the increased myocardial susceptibility to ischemia associated with obesity and metabolic syndrome involves a deregulated activation of the Rheb/mTORC1 pathway [67, 133].

The energy crisis caused by ischemia in the heart is at least partially resolved at the time of reperfusion, which further activates autophagy through mechanisms different from those involved in autophagy during ischemia [131]. We have reported that ROS generated in the mouse heart during reperfusion injury mediate the upregulation of Beclin1 [131]. In these experiments, Beclin1 was strongly upregulated during the reperfusion phase but not during the ischemic phase. These results suggest that strong induction of Beclin1 by ROS may play an important role in mediating the stimulation of autophagy during reperfusion.

Whether autophagy induced during reperfusion is beneficial or detrimental remains controversial. Although Hamacher-Brady et al. [135] showed that enhancing autophagic flux during ischemia/reperfusion protects against ischemic/reperfusion injury in cardiomyocytes, we found that autophagosome formation and cardiac ischemic injury are significantly attenuated in mice with heterozygous disruption of Beclin1 [131] and GSK-3*β* [132]. It is possible that exaggerated activation of autophagy during excessive ROS production may trigger cell death. In this regard, it has been recently proved the existence of a form of cell death named “autosis,” which is characterized by cellular nuclear convolution at early stages and by focal swelling of perinuclear space at late stages and which is blocked by autophagy inhibition or knockdown of the Na+/K+-ATPase *α*1 subunit, but not by inhibitors of apoptosis or necrosis [136].

Despite the controversy of whether autophagy activation is protective or detrimental during reperfusion injury, mitophagy activation appears to protect the heart during ischemia/reperfusion. Inhibition of Drp1 was found to promote ischemic injury in the heart through inhibition of fission and mitophagy (Ikeda Y and Sadoshima J, unpublished observations). Parkin inhibition increases cardiomyocyte death in response to hypoxia-reoxygenation* in vitro*, and parkin deletion* in vivo *abolished the cardioprotective effect of ischemic preconditioning [137]. In addition, simvastatin was shown to reduce cardiac damage in response to ischemia/reperfusion by activating mitophagy [138].

Autophagy inhibition negatively impacts cardiac remodeling after chronic myocardial infarction due to accumulation of misfolded proteins and mitochondrial dysfunction. We recently found that the serine/threonine kinase MST1 phosphorylates Beclin1 at threonine 108, thereby promoting its interaction with Bcl-2 and inhibiting autophagy. MST1-dependent inhibition of autophagy accelerates cardiac remodeling during chronic myocardial infarction [139]. Interestingly, parkin knockout mice display reduced mitophagy and survival after myocardial infarction, together with increased cardiac remodeling and accumulation of dysfunctional mitochondria [140]. These data suggest that mitophagy plays a crucial role in delaying cardiac remodeling after myocardial infarction.

Aging is associated with increased cardiac hypertrophy, and defects in autophagy promote cardiac hypertrophy [58, 59]. Cardiac deletion of Atg5 is associated with the development of eccentric hypertrophy over time [76]. We recently reported an experiment in which mice were subjected to transverse aortic constriction (TAC) for 1 week, after which the constriction was removed (DeTAC) [141]. Regression of cardiac hypertrophy was observed after DeTAC, accompanied by upregulation of autophagy in conjunction with upregulation of FOXO1. This regression of cardiac hypertrophy was significantly attenuated by inhibition of either autophagy or FOXO1. These results suggest that autophagy and FOXO1 play an essential role in mediating the regression of cardiac hypertrophy during mechanical unloading [141]. In addition, PINK1 knockout mice develop cardiac dysfunction and hypertrophy associated with oxidative stress, mitochondrial dysfunction, fibrosis, and apoptosis at baseline [142], indicating that mitophagy is important for inhibiting maladaptive hypertrophy. Interestingly, in* Drosophila,* inhibition of parkin leads to progressive cardiac dilation and dysfunction through inhibition of mitophagy, increased mitochondrial fusion and subsequent contagion of healthy mitochondria with damaged ones [143].

## 6. Perspectives

Translational research represents a stem of scientific research that helps make findings from basic science useful for practical applications that enhance human health and wellbeing. Deeply established with multidisciplinary collaboration, translational research has the enormous potential to move applied science forward. To date, this is particularly true in so-called “translational medicine” research that aims to move “from bench to bedside” or from laboratory experiments through clinical trials to point-of-care patient applications. In this context, mitochondrial autophagy and mitochondrial dynamics emerge to have an important role in regulating mitochondrial function and quality control. However, autophagy appears to be inhibited during aging [16]. In future studies, it will be important to investigate the relationship between aging and mitophagy and mitochondrial dynamics more deeply. In particular, it should be interesting to evaluate the molecular mechanisms and signaling pathway interactions through which aging affects mitophagy, mitochondrial fusion, and mitochondrial fission. This information would help in the generation of specific drugs that are capable of reactivating these mechanisms to limit age-dependent mitochondrial dysfunction and ROS accumulation. In support of the potential usefulness of reactivating mitophagy to increase longevity, it has been recently demonstrated that parkin overexpression extends life span in* Drosophila* [144]. Similar studies in mammals are strongly encouraged.

**Figure 4:**
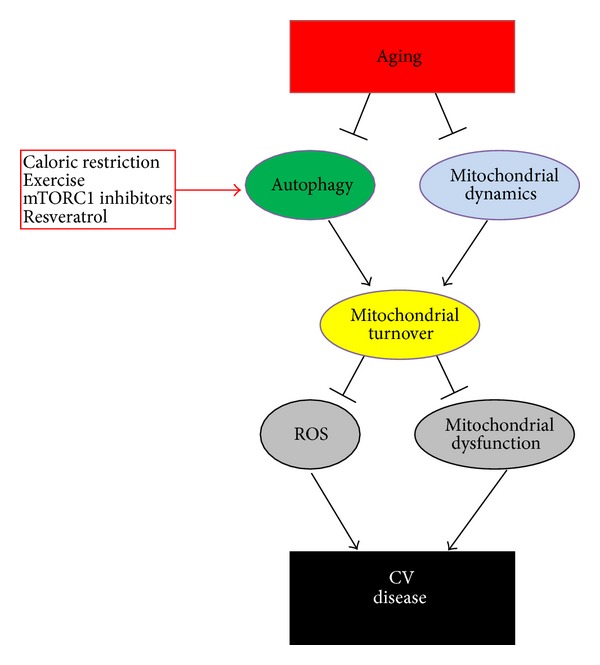
The importance of autophagy in the aged heart. Aging inhibits autophagy and affects mitochondrial dynamics. Autophagy inhibition is associated with accumulation of damaged mitochondria and oxidative stress, which favor the development of cardiovascular diseases.

Until specific pharmacological agents to target abnormalities in mitophagy and mitochondrial dynamics during aging are identified, the efficacy of reactivating general autophagy should be tested [19]. Autophagy would eliminate intracellular waste material and dysfunctional mitochondria that cause abnormal ROS generation during aging and cardiac derangements (Figure 4). Several interventions are known to induce autophagy and could be useful for promoting longevity. Caloric restriction, defined as a reduction in food intake without malnutrition, is a well-known antiaging intervention and physiological inducer of autophagy [16, 78]. Long-term caloric restriction inhibits the age-associated decline in diastolic function in the heart [84]. Pharmacological induction of autophagy may also effectively increase longevity. Metformin is an activator of AMPK that promotes autophagy, which contributes to its antidiabetic effect [19]. Of note, low-dose metformin was recently found to extend health span and life span in mice without metabolic disorders [145, 146]. Metformin administration increases insulin sensitivity, reduces cholesterol levels, improves physical performance, and exerts antioxidant and anti-inflammatory effects during the aging process [145]. Suppression of mTOR by rapamycin prevents cardiac senescence in conjunction with inhibition of lipofuscin accumulation in the heart [83, 85], and resveratrol is able to induce autophagy and increase longevity [147]. Exercise also induces autophagy in skeletal muscle, which contributes to the improvement in glucose tolerance induced by exercise in the context of a high-fat diet [148]. The actual role of autophagy in mediating the beneficial effects of these compounds on life span still requires clarification. Of note, excessive activation of autophagy would not necessarily be protective because it could cause depletion of essential proteins and organelles [19, 67–69]. Future studies should therefore also investigate the level to which it would be beneficial to reactivate autophagy in the aged heart. Notably, all these emerging data have obvious and nonnegligible theoretical and applicative implications: they start from a biological/molecular core that is made of novel observations, concepts, and tools and aim to reach wed cell biology with clinical connotations. Accordingly, based on this new view and taken altogether, these advances in cell biology may herald a new area of cardiovascular regenerative antiaging and personalized medicine in the upcoming years, exploiting this body of evidence as a long-missed benchmark for the development of approaches moving from insight to insight and back.
